# Impact of Switching From Immediate- or Prolonged-Release to Once-Daily Extended-Release Tacrolimus (LCPT) on Tremor in Stable Kidney Transplant Recipients: The Observational ELIT Study

**DOI:** 10.3389/ti.2024.11571

**Published:** 2024-04-17

**Authors:** Magali Giral, Philippe Grimbert, Baptiste Morin, Nicolas Bouvier, Matthias Buchler, Jacques Dantal, Valérie Garrigue, Dominique Bertrand, Nassim Kamar, Paolo Malvezzi, Karine Moreau, Yoni Athea, Yannick Le Meur

**Affiliations:** ^1^ CHU Nantes, Hotel Dieu, Nantes, France; ^2^ Hôpitaux Universitaires Henri Mondor, Créteil, France; ^3^ Chiesi SAS, Bois Colombes, France; ^4^ CHU Caen-Normandie, Caen, France; ^5^ CHRU Tours, Hôpital Bretonneau, Tours, France; ^6^ CHRU Montpellier, Hôpital Lapeyronie, Montpellier, France; ^7^ CHU Rouen, Hôpital Bois Guillaume, Rouen, France; ^8^ CHU Toulouse, Université Paul Sabatier Toulouse III, Toulouse, France; ^9^ CHU Grenoble, Hôpital Nord Michallon, Grenoble, France; ^10^ CHU Bordeaux, Pellegrin, Bordeaux, France; ^11^ CHU Brest, La Cavale Blanche, Brest, France

**Keywords:** extended-release tacrolimus, LCPT, immunosuppression, kidney transplantation, tremor, C_0_/D ratio, fast metabolizer, quality of life

## Abstract

Once-daily extended-release tacrolimus (LCPT) exhibits increased bioavailability versus immediate-release (IR-TAC) and prolonged release (PR-TAC) tacrolimus. Improvements in tremor were previously reported in a limited number of kidney transplant patients who switched to LCPT. We conducted a non-interventional, non-randomized, uncontrolled, longitudinal, prospective, multicenter study to assess the impact of switching to LCPT on tremor and quality of life (QoL) in a larger population of stable kidney transplant patients. The primary endpoint was change in The Essential Tremor Rating Assessment Scale (TETRAS) score; secondary endpoints included 12-item Short Form Survey (SF-12) scores, tacrolimus trough concentrations, neurologic symptoms, and safety assessments. Subgroup analyses were conducted to assess change in TETRAS score and tacrolimus trough concentration/dose (C_0_/D) ratio by prior tacrolimus formulation and tacrolimus metabolizer status. Among 221 patients, the mean decrease of TETRAS score after switch to LCPT was statistically significant (*p* < 0.0001 vs. baseline). There was no statistically significant difference in change in TETRAS score after switch to LCPT between patients who had received IR-TAC and those who had received PR-TAC before switch, or between fast and slow metabolizers of tacrolimus. The overall increase of C_0_/D ratio post-switch to LCPT was statistically significant (*p <* 0.0001) and from baseline to either M1 or M3 (both *p <* 0.0001) in the mITT population and in all subgroups. In the fast metabolizers group, the C_0_/D ratio crossed over the threshold of 1.05 ng/mL/mg after the switch to LCPT. Other neurologic symptoms tended to improve, and the SF-12 mental component summary score improved significantly. No new safety concerns were evident. In this observational study, all patients had a significant improvement of tremor, QoL and C_0_/D ratio post-switch to LCPT irrespective of the previous tacrolimus formulation administered (IR-TAC or PR-TAC) and irrespective from their metabolism status (fast or slow metabolizers).

## Graphical Abstract

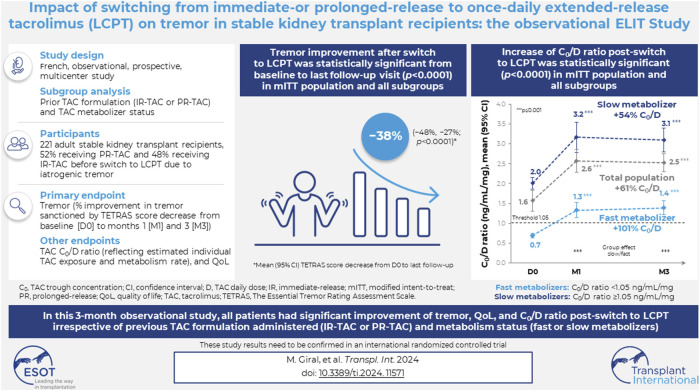

## Introduction

Tacrolimus is currently the mainstay of immunosuppressive treatment in kidney transplant recipients [1, 2], and its use has contributed to improved 1-year graft survival rates, which are now approximately 95%–98% [3]. However, due to its narrow therapeutic range, strict monitoring of tacrolimus trough blood concentrations is required, as drug overexposure is often associated with increased toxicities, while underexposure may lead to graft rejection [4].

Calcineurin inhibitors (CNIs), including tacrolimus, are commonly associated with neurotoxicity [5, 6]. Because of their frequency and severity, neurologic symptoms are an important factor in morbidity and impaired quality of life (QoL) in kidney transplant recipients. One of the most frequently reported and disabling neurologic symptoms is tremor (observed in 34%–54% of tacrolimus recipients) [7, 8]. Although the pathogenesis is unknown, some observations suggest that the occurrence and severity of neurologic symptoms are correlated with tacrolimus plasma concentrations [9–11].

Tacrolimus is available as three formulations, each exhibiting a specific pharmacokinetic profile: immediate-release tacrolimus (IR-TAC), prolonged-release tacrolimus (PR-TAC), and extended-release tacrolimus (LCPT) [12].

LCPT has been developed using the MeltDose™ (Veloxis Pharmaceuticals) drug delivery technology that improves drug solubility and, thus, absorption. This feature, combined with a more distal release in the gastrointestinal tract, results in a significant increase in tacrolimus bioavailability with LCPT compared with IR-TAC and PR-TAC, an improvement in trough concentration/dose (C_0_/D) ratio (trough tacrolimus blood concentration normalized by daily dose, which reflects estimated individual tacrolimus exposure and metabolism rate) [13], and may significantly reduce the maximum plasma concentration (C_max_) [14] and/or the peak-to-trough fluctuations in blood drug concentrations [12, 14]. Hence, a 30% decrease in the daily dose required to achieve a similar systemic tacrolimus exposure and clinical efficacy has been observed with LCPT versus IR-TAC [14, 15]. In addition, LCPT has been shown to be at least as effective as IR-TAC in stable kidney transplant patients [16, 17], or as IR-TAC and PR-TAC in newly transplanted patients [18, 19], as measured by treatment failure rates at 6 and 12 months. The pre-dose concentration to daily dose (C_0_/D) ratio of tacrolimus seems to be an appropriate tool for identifying patients at risk of developing calcineurin-inhibitor toxicity such as rejection and lower renal function with increased risk of poor outcome after kidney transplantation [20–22]. A low tacrolimus concentration/dose ratio has been shown to increase the risk for the development of acute calcineurin inhibitor-induced nephrotoxicity [23].

The 7-day STRATO study of 38 stable kidney transplant recipients suggested that a switch in tacrolimus formulation from IR-TAC to LCPT resulted in a significant reduction in drug-induced tremor and a significant improvement in QoL [24]. The ELIT (*Evolution à Long terme des tremblements Iatrogènes de Tacrolimus* or Long-term Outcomes of Tacrolimus-induced Tremor) study was conducted, under real-life conditions, to further investigate whether kidney transplant patients may benefit from LCPT treatment, in terms of tremor improvement, tacrolimus dose reduction, C_0_/D ratio improvement, clinical response, QoL, and safety. The primary study objective was to assess the change in tremor and the impact on daily activities after switching to LCPT.

## Materials and Methods

### Study Design

The ELIT study was a non-interventional, non-randomized, uncontrolled, longitudinal, prospective, multicenter study that was conducted at 25 hospitals performing kidney transplants in France. The study was approved by the French Authority for computerized research data (Comité Consultatif sur le Traitement de l’Information en Matière de Recherche dans le domaine de la Santé, C.C.T.I.R.S.) and all subjects provided written consent for the use of their data for the purpose of this study.

### Participants

Eligible patients were aged >18 years, had undergone their first kidney transplant <7.5 years prior to the study, had stable kidney function, had received tacrolimus for ≥8 weeks with the dose unchanged for ≥15 days, had tacrolimus trough blood concentrations of 4–15 ng/mL, and were presenting with tremor requiring treatment adjustment and had switched from IR-TAC or PR-TAC to LCPT, according to clinician judgement. Patients diagnosed with Parkinson’s disease or any other neurologic syndrome potentially associated with tremor were excluded.

### Treatment

Patients were treated at their attending clinician’s discretion, and in accordance with product labelling. As such, no constraints were imposed on the dosages and administration schedules. The practical modalities of the switch to LCPT were also conducted at the discretion of the attending clinician.

Tacrolimus daily doses, trough blood concentrations, and any dosage adjustments were reported at each assessment (see below). In the event of treatment discontinuation, the date and the reason(s) for discontinuation were specified.

### Outcomes and Assessments

All data were collected by each investigational site and recorded in an electronic case report form at three visits: baseline/Day 0 (D0), Month 1 (M1), and Month 3 (M3). Baseline/D0 corresponds to the day of switching from IR-TAC or PR-TAC to LCPT.

The primary endpoint was the percent improvement in The Essential Tremor Rating Assessment Scale (TETRAS) score [25] from baseline to the last follow-up visit. TETRAS scores were obtained at each study visit. This scale comprises 12 items, each scored from 0 to 4, and assesses the impact of tremors on a patient’s activities of daily living. The total TETRAS score (ranging from 0 to 48) is the sum of the 12 items, with higher scores indicating more severe tremors.

The key secondary endpoint was patient health-related QoL, assessed using the 12-item Short Form Survey (SF-12) [26] at D0 and M3. The SF-12 is a 12-item questionnaire, providing two composite scores: a “physical component summary” score (including “physical functioning,” “role-physical,” “bodily pain” and ”general health perceptions” scores) and a “mental component summary” score (including “vitality,” “role-emotional,” “social functioning,” and “mental health” scores). All scores are standardized on a 0 to 100 scale, with 0 indicating the worst QoL.

Patient demographic and clinical characteristics were collected at baseline. At each visit, blood tacrolimus concentrations, LCPT dose, and neurologic symptoms were recorded, and standard safety assessments were conducted (e.g., adverse events [AEs] and laboratory tests, including blood cell count, biochemistry, liver function, kidney function, and lipid profile). C_0_/D ratio was calculated for each patient by dividing the tacrolimus pre-dose concentration (C0) by the corresponding daily tacrolimus dose (D). Patients were categorized into two metabolizer groups based on a cut-off value of 1.05 ng/mL/mg at baseline: patients with a tacrolimus C_0_/D ratio <1.05 ng/mL/mg were defined as fast metabolizers, while patients with a C_0_/D ratio ≥1.05 ng/mL/mg were defined as slow metabolizers.

Patients were also categorized into two analysis subgroups: patients treated with IR-TAC as the last tacrolimus formulation prior to the switch to LCPT (the IR-TAC pretreated group) and patients treated with PR-TAC as the last tacrolimus formulation prior to the switch to LCP (the PR-TAC pretreated group).

### Statistical Analyses

It was estimated that a total of 229 patients would be required to detect a change of ≥15% on the TETRAS scale, with an alpha risk of 5% and a beta risk of 10%, assuming a standard deviation (SD) of 70% for the improvement rate from baseline to the last follow-up visit. To account for 15% of observations being unusable or missing, it was estimated that data from 270 patients were required.

The efficacy analyses were performed on the modified intent-to-treat (mITT) population, which included all patients with at least one efficacy assessment. All patients who received at least one dose of LCPT were included in the safety analysis population.

Descriptive statistics were summarized as mean with SD, minimum, maximum, and median with interquartile range (IQR) for qualitative data, and number of patients with percentages for quantitative data. All statistical tests were carried out at a two-sided, 5% significance level.

Total TETRAS scores were calculated if at least half of the 12 items were completed, and missing items were replaced with the average of the items completed. The mean change from baseline was presented with 95% confidence intervals (CIs) at each available visit. The primary endpoint was the change from baseline to the last follow-up visit (M3, or M1 if M3 not available). The overall change over time in TETRAS scores was evaluated using a repeated measures analysis of variance, with time as the fixed effect and patient as the random effect; TETRAS scores at M1, M3, or the last follow-up visit were compared with the baseline score using Dunnett’s test. Subgroups were compared by an analysis of covariance for change in TETRAS scores at M1 and M3 versus baseline. The same analyses (mean change from baseline, overall change over time, and comparison of values at M1 or M3 vs. baseline) were performed for tacrolimus trough blood concentrations and C_0_/D ratio. The mean change in C_0_/D ratio from baseline to M1 and M3 was compared in subgroups using the Wilcoxon test. The association between TETRAS scores and tacrolimus trough blood concentrations was assessed using Spearman’s rank correlation.

The mean change in SF-12 scores from baseline to M3 was presented for patients with evaluable data; SF-12 scores at M3 were compared with baseline using the Wilcoxon test for SF-12 individual scores and the Student t-test for SF-12 composite scores.

Laboratory parameters were summarized with descriptive statistics. Estimated glomerular filtration rate (eGFR) was calculated using the Chronic Kidney Disease Epidemiology Collaboration (CKD-EPI) equation [27].

Statistical analyses were performed using SAS^®^ software (version 9.4).

## Results

### Participants

**FIGURE 1:**
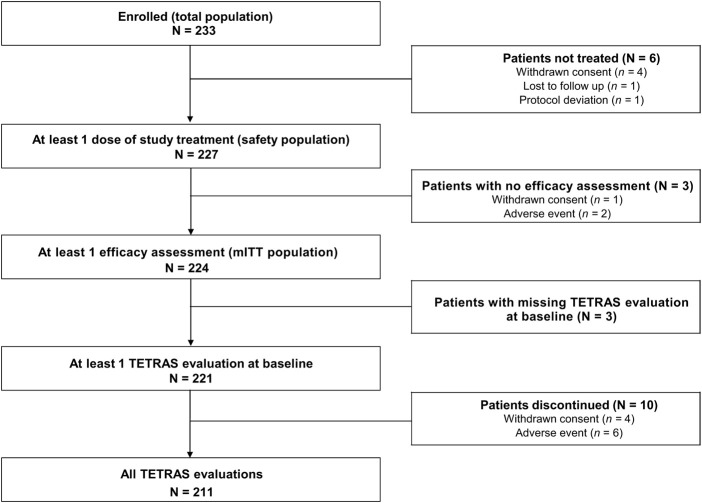
Population flow chart. *Three patients were excluded from the primary endpoint analyses (one patient had no evaluable TETRAS data on Day 0, and two patients had no post-baseline TETRAS data); therefore, the primary endpoint analysis was performed using data from 221 patients. mITT, modified intent-to-treat; TETRAS, The Essential Tremor Rating Assessment Scale.

Over an 18-month period (15 June 2017 to 31 December 2018), 233 patients were recruited. Among these, 227 were included in the safety population, and 224 in the mITT efficacy population. Three patients had missing TETRAS evaluation at D0, and 10 patients had missing TETRAS evaluation at M1 or M3. Thus, TETRAS score analyses have been made on 221 patients at baseline and 211 patients at M1 and M3 (Figure 1).

In the mITT population, 57.6% of patients were male, and the median (IQR) age was 58 (46.0–67.5) years. The median (IQR) time from kidney transplantation to the switch to LCPT was 11.02 (4.75–28.77) months. The baseline demographic and disease-related characteristics of the mITT population are shown in Table 1.

**TABLE 1:** Patient demographics and disease-related characteristics at baseline (study population and modified intent-to-treat population).

Characteristic/demographic	Total mITT population (*N* = 224)
Male sex, *n* (%)	129 (57.6)
Median (IQR) age at enrolment, years	58.0 (46.0–67.5)
Initial cause of nephropathy, *n* (%)	
Polycystic kidney disease	44 (22.4)
Glomerulopathy	34 (17.3)
Diabetic nephropathy	17 (8.7)
Immunoglobulin A nephropathy	16 (8.2)
Hypertensive nephropathy	14 (7.1)
Vascular nephropathy	14 (7.1)
Interstitial nephropathy	12 (6.1)
Congenital nephropathy	11 (5.6)
Other	34 (17.3)
Dialysis received before transplant, *n* (%)	188 (83.9)
History of diabetes, *n* (%)	58 (25.9)
Median (IQR) time from transplant to LCPT switch, months	11.02 (4.8–28.8)
Post-transplantation treatment other than tacrolimus, *n* (%)	*n* = 222
	219 (98.6)
Antibiotics	219 (98.6)
Corticoids received post-transplant	144 (64.9)
Immunosuppressor other than tacrolimus	157 (70.7)
Induction (ATG or immunoglobulin)	99 (44.6)
Deceased donor, *n* (%)	185 (82.6)
Tacrolimus formulation at baseline (before the switch), *n* (%)	224 (100)
Prolonged-release tacrolimus (PR-TAC)	117 (52.2)
Advagraf^®^ (PR-TAC)	117 (52.2)
Immediate-release tacrolimus (IR-TAC)	107 (47.8)
Prograf^®^ (IR-TAC)	98 (43.8)
Adoport^®^ (IR-TAC)	7 (3.1)
Modigraf^®^ (IR-TAC)	2 (0.9)
Median (IQR) time since tremor onset, months	*n* = 172
	5.9 (2.3–17.9)
At least one other neurological symptom, *n* (%)	122 (54.5)
Serum creatinine, µmol/L	
Mean (SD)	139.6 (44.1)
Min; max	45.0; 321.0
eGFR, mL/min/1.73m^2^	
Mean (SD)	48.6 (18.5)
Min; Max	16.8; 113.9

eGFR, estimated glomerular filtration rate; LCPT, extended-release tacrolimus; IQR, interquartile range; IR-TAC, immediate-release tacrolimus; PR-TAC, prolonged-release tacrolimus; SD, standard deviation.

Before switching to LCPT, 117 (52.2%) patients were receiving PR-TAC and 107 (47.8%) patients were receiving IR-TAC. Of the PR-TAC pretreated patients, 58.1% were male versus 57.0% of IR-TAC pretreated patients. The median (IQR) age was 56 (45.0–66.0) years in PR-TAC pretreated patients versus 61.0 (48.0–70.0) years in IR-TAC pretreated patients. The median (IQR) time from kidney transplantation to the switch to LCPT was 17.25 (6.10–31.74) months in PR-TAC pretreated patients versus 6.66 (4.03–15.34) months in IR-TAC pretreated patients.

Based on the C_0_/D ratio cut-off value of 1.05 ng/mL/mg, 73 (33.8%) patients were characterized as fast metabolizers and 143 (66.2%) as slow metabolizers. Of the fast metabolizer patients, 56.2% were male versus 59.4% of the slow metabolizer patients. The median (IQR) age was 53 (42.0–60.0) years in fast metabolizer patients versus 61.0 (49.0–70.0) years in slow metabolizer patients. The median (IQR) time from kidney transplantation to the switch to LCPT was 9.18 (4.62–26.82) months in the fast metabolizers versus 11.54 (4.82–29.93) months in the slow metabolizers.

### Primary Endpoint (Tremor)

**FIGURE 2:**
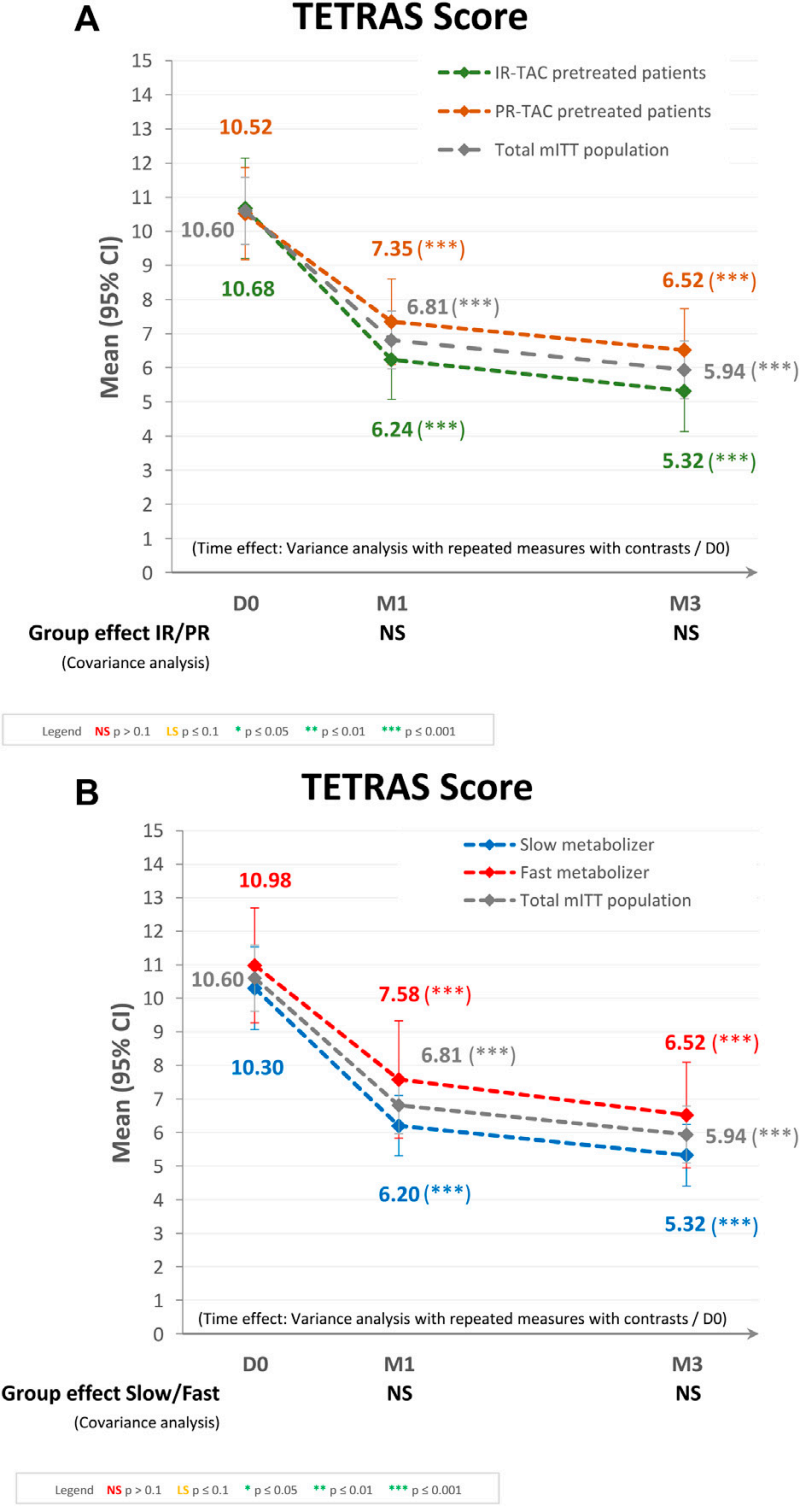
Tremor evaluation using the TETRAS score after switching to LCPT in the modified intent-to-treat (mITT) population: **(A)** IR-TAC pretreated patients versus PR-TAC pretreated patients, and **(B)** fast metabolizer patients versus slow metabolizer patients. CI, confidence interval; D, day; IR-TAC, immediate-release tacrolimus; M, month; NS, not significant; PR-TAC, prolonged-release tacrolimus; TETRAS, The Essential Tremor Rating Assessment Scale.

The primary endpoint analysis included data from the 221 patients. The mean (95% CI) total TETRAS scores obtained at D0, M1, and M3 were 10.60 (9.61, 11.58), 6.81 (5.96, 7.67), and 5.94 (5.79, 6.79), respectively for the mITT population (Figure 2A). The overall decrease in TETRAS score over time for the mITT population was statistically significant (*p* < 0.0001), as were the decreases from baseline to either M1 or M3 (both *p* < 0.0001). The mean (95% CI) change in TETRAS score from baseline was −28.30% (−39.00%, −17.60%) at M1 and −38.68% (−49.77%, −27.60%) at M3. These results were confirmed by the primary endpoint analysis, with a mean (95% CI) change in TETRAS score from baseline to last follow-up visit of −37.63% (−48.32%, −26.95%; *p* < 0.0001). When categorized by change in TETRAS score, 151 patients (71.6%) at M1 and 163 (77.3%) at M3 were classified as “improved,” 23 (10.9%) at M1 and 12 (5.7%) at M3 had “no change,” and 37 (17.5%) at M1 and 36 (17.1%) at M3 were classified as “worsened.”

Regarding the subgroup analysis by pretreatment (IR-TAC pretreated vs. PR-TAC pretreated), the mean (95% CI) total TETRAS scores obtained at D0, M1, and M3 were 10.52 (9.17, 11.87), 7.35 (6.10, 8.60), and 6.52 (5.31, 7.73), respectively, for PR-TAC pretreated patients and 10.68 (9.21, 12.14), 6.24 (5.07, 7.42), and 5.32 (4.13, 6.52), respectively, for IR-TAC pretreated patients. The overall decrease in TETRAS score after the switch was statistically significant (*p* < 0.0001) in the two groups, as were the decreases from baseline to either M1 or M3 (both *p* < 0.0001). There was no statistically significant difference between the two groups (IR-TAC pretreated patients and PR-TAC pretreated patients) in terms of change in TETRAS score from baseline to either M1 or M3 (Figure 2A).

Regarding the subgroup analysis by tacrolimus metabolizer status (fast metabolizers vs. slow metabolizers), the mean (95% CI) total TETRAS scores observed at D0, M1, and M3 were 10.98 (9.27, 12.68), 7.58 (5.83, 9.34), and 6.52 (4.95, 8.09), respectively, for the fast metabolizer group and 10.30 (9.07, 11.54), 6.20 (5.30, 7.09), and 5.32 (4.40, 6.23), respectively, for the slow metabolizer group. The overall decrease in TETRAS score after the switch was statistically significant (*p* < 0.0001) in the two groups, as were the decreases from baseline to either M1 or M3 (both *p* < 0.0001). There was no statistically significant difference between the two groups (fast metabolizers and slow metabolizers) in terms of change in TETRAS score from baseline to either M1 or M3 (Figure 2B).

### Secondary Endpoints

#### Tacrolimus Dose and Trough Concentration

At baseline, the mean dose of tacrolimus (irrespective of the formulation) was 0.113 mg/kg/day. After switching to LCPT, the mean dose of tacrolimus was 0.071 mg/kg/day (4.89 mg/day); it was 0.067 mg/kg/day (4.60 mg/day) at M1 and 0.062 mg/kg/day (4.29 mg/day) at M3. While the mean tacrolimus dose decreased over time, the mean (95% CI) trough blood concentration increased from 7.04 (6.79, 7.29) ng/mL at D0 to 7.81 (7.45, 8.16) ng/mL at M1 and 7.59 (7.27, 7.92) ng/mL at M3. The mean (SD) change in trough blood concentration from baseline was +0.73 (3.09) ng/mL at M1 (*p* = 0.0005) and +0.55 (2.65) ng/mL at M3 (*p* = 0.0103). The overall increase in trough blood concentration over time was statistically significant (*p* = 0.0006).

There was no correlation between the change in tacrolimus trough blood concentration from D0 to M1 and the change in TETRAS score (Spearman’s *ρ* = −0.02).

#### Trough Concentration/Dose (C_0_/D) Ratio

**FIGURE 3:**
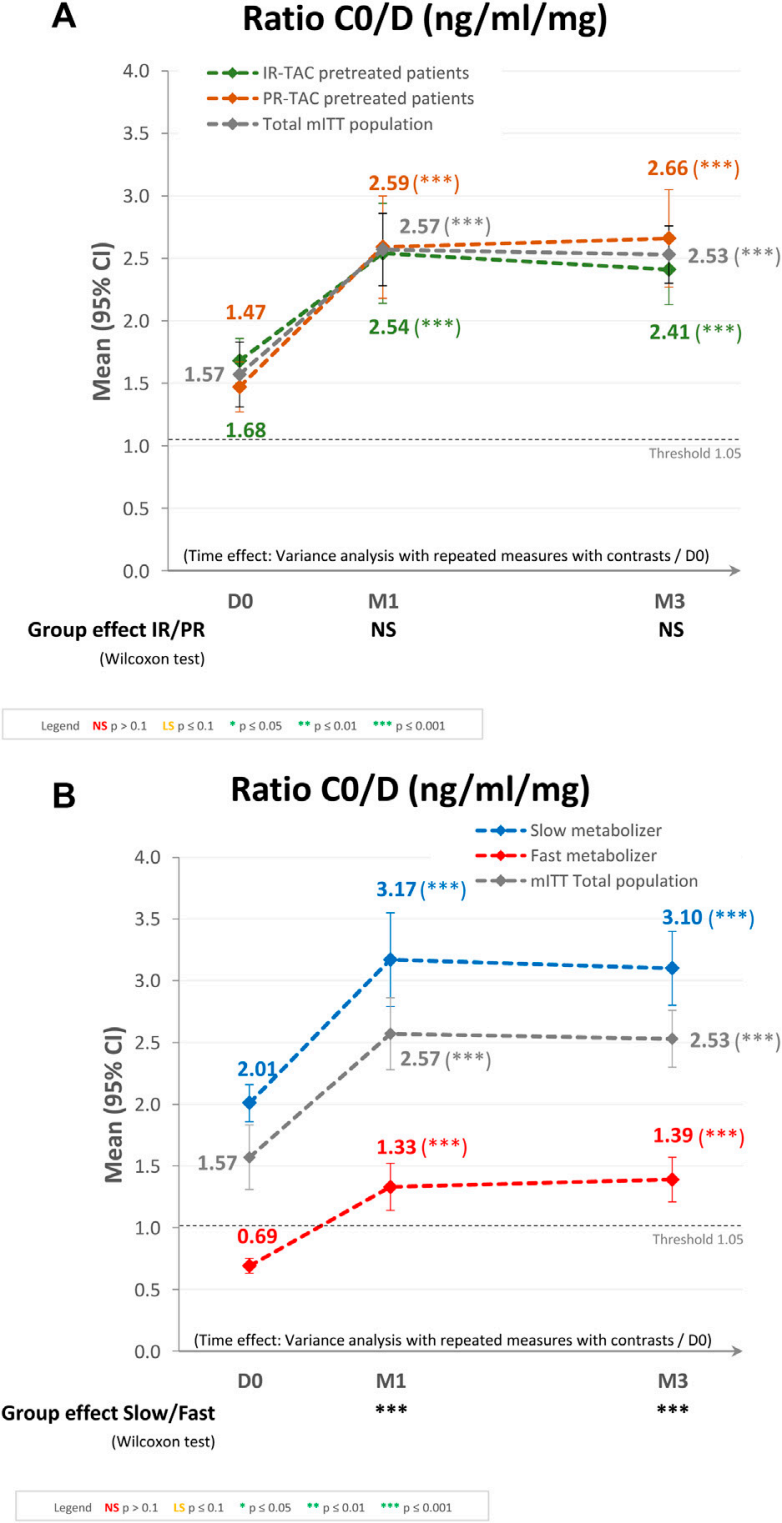
Trough concentration/dose (C_0_/D) ratio after switching to LCPT in the modified intent-to-treat (mITT) population: **(A)** IR-TAC pretreated patients versus PR-TAC pretreated patients; and **(B)** fast metabolizer patients versus slow metabolizer patients. CI, confidence interval; D, day; IR-TAC, immediate-release tacrolimus; M, month; NS, not significant; PR-TAC, prolonged-release tacrolimus.

Regarding the subgroups analysis by pretreatment (IR-TAC pretreated vs. PR-TAC pretreated), the mean (95% CI) C_0_/D ratios observed at D0, M1, and M3 were 1.47 (1.27, 1.67), 2.59 (2.18, 3.00), and 2.66 (2.27, 3.04) ng/mL/mg, respectively, for the PR-TAC pretreated group and 1.68 (1.50, 1.86), 2.54 (2.14, 2.95), and 2.41 (2.13, 2.68) ng/mL/mg, respectively, for the IR-TAC pretreated group. The overall increase in C_0_/D ratio post-switch to LCPT was statistically significant (*p* < 0.0001) in the two groups, and from baseline to either M1 or M3 (both *p* < 0.0001). However, there was no statistically significant difference in terms of C_0_/D ratio between the two groups (Figure 3A).

Regarding the subgroups analysis by tacrolimus metabolizer status (fast metabolizers vs. slow metabolizers), the mean (95% CI) C_0_/D ratios observed at D0, M1, and M3 were 0.69 (0.63, 0.74), 1.33 (1.14, 1.51), and 1.39 (1.21, 1.57) ng/mL/mg, respectively, for the fast metabolizer group and 2.01 (1.86, 2.16), 3.17 (2.79, 3.55), and 3.10 (2.80, 3.40) ng/mL/mg, respectively, for the slow metabolizer group. The overall increase in C_0_/D ratio post-switch to LCPT was statistically significant (*p* < 0.0001) in the two groups, and from baseline to either M1 or M3 (both *p* < 0.0001). In the fast metabolizer group, the C_0_/D ratio crossed over the threshold of 1.05 ng/mL/mg after the switch to LCPT. Furthermore, the difference between the two groups in terms of C_0_/D ratio at M1 and M3 was statistically significant (*p* < 0.0001; Figure 3B).

#### Quality of Life

**FIGURE 4:**
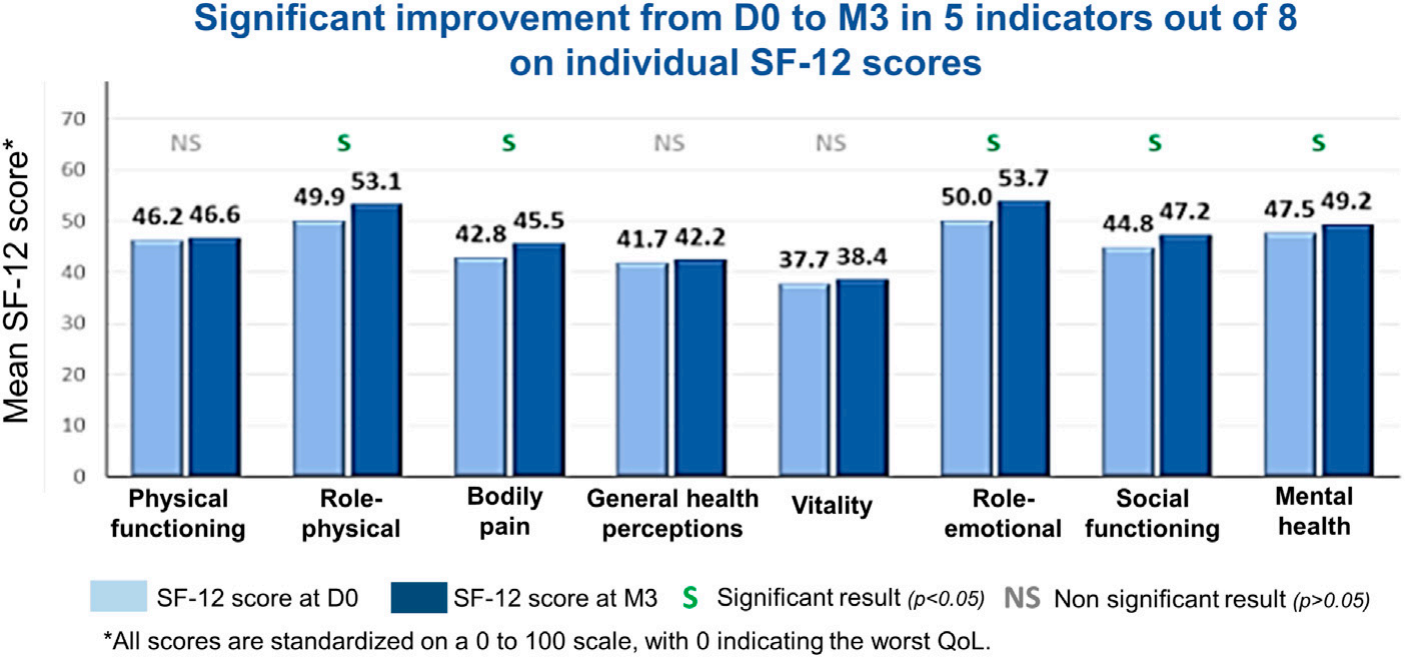
Mean 12-item Short Form Survey (SF-12) scores from baseline to Month 3 (M3) in the modified intent-to-treat population. D0, Day 0 (baseline); QoL, quality of life.

There was a statistically significant improvement from baseline in the individual SF-12 component scores of role-physical (*p* = 0.0001), bodily pain (*p* = 0.0019), role-emotional (*p* < 0.0001), social functioning (*p* = 0.0069), and mental health (*p* = 0.0197), as well as in the mental component summary scores (*p* = 0.0002). The improvement in the physical component summary score approached statistical significance (*p* = 0.0707; Table 2; Figure 4).

**TABLE 2:** Mean 12-item Short Form Survey (SF-12) scores over time (modified intent-to-treat population).

SF-12 component	*n*	Mean (SD) SF-12 score			*p*-value^a^
		Day 0	Month 3	Change from baseline	
Physical functioning	199	46.2 (9.9)	46.6 (10.4)	3.7 (26.8)	0.6604
Role-physical	200	49.9 (12.4)	53.1 (11.6)	3.2 (12.4)	**0.0001**
Bodily pain	199	42.8 (11.8)	45.5 (11.5)	2.7 (11.2)	**0.0019**
General health perceptions	198	41.7 (11.1)	42.2 (10.9)	0.5 (9.9)	0.6252
Physical component summary	194	44.4 (9.5)	45.5 (9.6)	1.2 (8.9)	0.0707
Vitality	200	37.7 (12.4)	38.4 (11.9)	0.7 (11.0)	0.2907
Role-emotional	198	50.0 (14.2)	53.7 (12.7)	3.7 (12.3)	**<0.0001**
Social functioning	200	44.8 (11.1)	47.2 (10.7)	2.4 (12.1)	**0.0069**
Mental health	200	47.5 (13.0)	49.2 (12.6)	1.7 (11.3)	**0.0197**
Mental component summary	194	46.1 (13.0)	48.8 (11.3)	2.7 (10.0)	**0.0002**

^a^ Significant *p*-values are shown in bold. Mean difference from baseline was evaluated statistically using the Wilcoxon test, except for the physical component summary and the mental component summary, for which the Student’s t-test was used.

SD, standard deviation.

#### Other Neurologic Symptoms

The overall number of patients with at least one post-baseline evaluation and one other neurologic symptom decreased from 121 (54.8%) at D0 to 103 (48.1%) at M1 and 83 (39.2%) at M3. All assessed neurologic symptoms reported at baseline had decreased in frequency by M1; subsequently, all but nightmares and photophobia decreased in frequency between M1 and M3. Although all neurologic symptoms decreased in frequency from D0 to M3, those symptoms reported in >15% of patients at D0 (i.e., headaches, insomnia, paresthesia/dysesthesia, and blurred vision) were still present in >10% of patients at M3.

#### Kidney Function and Other Laboratory Parameters

Kidney function was unchanged during the study: mean (SD) serum creatinine levels were 140.01 (44.71), 144.70 (49.54), and 143.30 (46.98) µmol/L at D0, M1, and M3, respectively. Mean (SD) eGFR values were 48.54 (18.59), 47.49 (18.94), and 47.54 (18.58) mL/min/1.73 m^2^ at D0, M1, and M3, respectively. Other renal function parameters (creatinine clearance) were numerically similar between study time points (data not shown).

There were no notable differences in lipid profiles during the study, including total cholesterol, high-density lipoprotein cholesterol, and triglyceride levels. There were also no notable changes over time in other laboratory parameters (blood cell count, blood glucose, liver enzyme, proteinemia/proteinuria).

### Adverse Events

During the 3-month follow-up, 117 patients (51.5%) presented with at least one AE and 43 (18.9%) with at least one treatment-related AE; 14 patients (6.2%) discontinued treatment due to AE(s), of whom eight discontinued due to a treatment-related AE. Serious AEs (SAEs) were reported in 39 patients (17.2%). Seven SAEs in six patients were considered to be related to LCPT (pneumocystis, hypertension, thrombotic microangiopathy, BK virus replication, basal cell carcinoma, epidermoid carcinoma, and cytomegalovirus infection). Three patients experienced a SAE considered unrelated to LCPT treatment that was fatal [pneumonia, head trauma (fall), and suicide].

### Graft Rejection

Two humoral graft rejections were reported (humoral rejection and chronic active humoral rejection): one case of humoral rejection for which biopsy confirmed the rejection but it was considered not related as the patient already presented with donor-specific antibodies on the day of graft (at a mean fluorescence intensity of 1470); and one case of chronic active humoral rejection (biopsy performed BANFF 2015 category 2). There was another case of acute renal failure that was also considered as suspicion of graft rejection; a biopsy was planned following an increase in creatinine but was cancelled as the levels returned to normal.

## Discussion

In the ELIT study, statistically significant decreases in mean total TETRAS scores were observed in patients switching from IR-TAC or PR-TAC to once-daily LCPT (−37.63% from switch to last follow-up visit; *p* < 0.0001), irrespective of the previous tacrolimus formulation administered and metabolism status (fast vs. slow metabolizers), suggesting tremor improvement in kidney transplant patients. These results—in a larger population—are in line with those of the STRATO study [24].

No correlation between tacrolimus trough blood concentrations and TETRAS scores was shown; however, the improvements in TETRAS scores were observed despite an increase in tacrolimus trough blood concentrations, suggesting that other pharmacokinetic parameters, such as tacrolimus peak blood concentrations (which were not evaluated in the current real-world study) or the C_0_/D ratio improvement (as shown by the results of this study), may play a role in reducing the incidence of tacrolimus-induced tremor. Moreover, we had to enlarge the predefined trough concentration range from 4–8 to 4–15 ng/mL to facilitate inclusion, and we had to extend the study enrollment period (from 12 to 18 months; protocol amended). Nevertheless, 73.2% of study participants (164 of 224 patients) had a tacrolimus trough concentration between 4 and 8 ng/mL at baseline (last dosage before inclusion): four patients had a trough concentration <4 ng/mL and 55 patients had a trough concentration between 8 and 13.90 ng/mL.

Previous studies examining a possible correlation between the pharmacokinetic characteristics of tacrolimus and the development of neurotoxicity have shown inconsistent results. More severe CNI-related toxicities have been reported with a higher CNI C_max_ [24, 28]; this may explain why the TETRAS scores in this study improved following the switch to LCPT, which has a consistently lower C_max_ than all other tacrolimus formulations [14]. Although neurologic symptom reduction was not correlated with tacrolimus trough blood concentrations [24], neurologic symptom reduction has been observed after discontinuation of tacrolimus or a decrease in dose [29]. Our study suggests that LCPT is associated with a different profile of neurologic effects compared with IR-TAC or PR-TAC and highlights the need for mechanistic studies to improve understanding of the pathophysiology of neurologic adverse effects that consider differences in the pharmacokinetic characteristics (including peak and trough blood concentrations) of different tacrolimus formulations.

In the ELIT study, the initial dose of LCPT was 37.1% lower than the dose of IR-TAC or PR-TAC administered prior to the switch, and the LCPT dose was reduced at each study visit; however, the tacrolimus trough blood concentration increased significantly over time. The tacrolimus C_0_/D ratio significantly improved post-switch to LCPT for all patients, irrespective of the previous tacrolimus formulation administered (IR-TAC or PR-TAC) and irrespective of the patients’ metabolism status (fast or slow metabolizers of tacrolimus). We can make the hypothesis that the improvement of tremors and neurologic symptoms after switch to LCPT can be explained by the C_0_/D ratio improvement. Previous studies have already shown that switching to LCPT increased tacrolimus bioavailability, C/D ratio, and was associated with a noticeable recovery of renal function in fast metabolizers [22].

These results are consistent with previous reports using the MeltDose^®^ technology, which demonstrated an increased bioavailability of LCPT compared with twice-daily formulations of tacrolimus [12, 14, 16] and with PR-TAC [30]. A comparative pharmacokinetic study of IR-TAC, PR-TAC, and LCPT formulations in stable renal transplant recipients demonstrated that there were significant differences between LCPT and both IR-TAC and PR-TAC, and that the formulations are not interchangeable with LCPT [12]. Based on the results of the ELIT study and exposure normalization analysis, a 36% total daily dose reduction is observed when converting from PR-TAC to LCPT and a 30% total daily dose reduction when converting from IR-TAC to LCPT. It is noteworthy that after the switch to LCPT, patients still had therapeutic drug exposure, despite the decreased dose. Further, the doses at each time point (D0, M1, and M3) were 59.7%–64.4% lower than that of the doses administered prior to the switch to LCPT. Interestingly, this is considerably less than the dosing conversion (1:0.7 on a mg:mg basis) outlined in the LCPT prescribing information [31], although it should be noted that the patients included in the current study received high tacrolimus doses at baseline and were experiencing tremors at baseline.

The switch to LCPT appeared to be associated with improvements in patient health-related QoL. We found statistically significant improvements from D0 to M3 in five of the eight individual components of the SF-12, as well as in the mental component summary. There was also an improvement, albeit not statistically significant, in the physical component summary. The observed clinical improvement in other neurologic symptoms is also likely to have been associated with this effect on QoL. Further, LCPT has been shown to improve psychomotor speed compared with cyclosporine [28], an effect that may also positively impact QoL.

No new efficacy or safety concerns were observed, including no clinically significant change in kidney function. This is consistent with evidence from liver or kidney transplant patients, which indicates that LCPT had less adverse impact on kidney function than the twice-daily tacrolimus formulation [13]. Switching to LCPT increased the bioavailability of tacrolimus and concentration-to-dose ratio, and was associated with a noticeable recovery of renal function in fast metabolizers [22]. It has been suggested that this reduced kidney toxicity may be due to a reduced peak tacrolimus concentration, in addition to improved bioavailability and reduced trough blood concentrations, after conversion to LCPT [13].

In the current study, the incidence of AEs (51.1% of patients had ≥1 AE; 18.9% had ≥1 treatment-related AE) and SAEs (17.2%) was higher than in the previous STRATO trial, in which 19.5% of patients experienced an AE, 2.4% a treatment-related AE, and no SAEs were reported [24]. This may be related to differences in study design (including duration), patient population, and sample size. STRATO was an open-label, multicenter, prospective, phase IIIb study, in which 38 stable kidney transplant patients with tremor were converted from twice-daily tacrolimus to once-daily LCPT and followed during the 7 days post-switch. In addition, the incidence of AEs in the ELIT study was lower than that reported by Budde et al. from a phase IV, randomized, open-label, parallel group study conducted in 10 European countries [19]. In that study of 200 patients over a 6-month period, 97.5% of patients had any AE, 36.5% had treatment-emergent adverse drug reactions, and 49.5% had an SAEs [19]. Further, in the LCPT international phase III study (double blind, randomized trial, 1-year follow-up; *n* = 268), 98.1% of study participants reported ≥1 AE and 61.9% reported ≥1 SAE [18], while in the LCPT phase III MELT study (two-armed, parallel group, prospective, randomized, open-label, multicenter, controlled, noninferiority trial; *n* = 162), 83.3% of patients had treatment-emergent AEs and 22.2% had a SAE [16]. The differences in the incidence of AEs in the ELIT study compared with these studies can be explained by the observational design of the ELIT study (generally less AEs reported). The incidence of AEs in the ELIT study was similar to that reported in the Spanish Better study (61.7% of patients experienced an AE and 27.1% experienced a SAE) [32], which had a similar study design (multicenter, prospective, observational; *n* = 133) to the ELIT study.

LCPT may offer a therapeutic alternative to other tacrolimus formulations, such as IR-TAC and PR-TAC, and allow for adequate balance between immunosuppression and adverse effects, given the large interpatient variability in tacrolimus bioavailability and absorption rates. This could be particularly relevant for patients who experience lower tacrolimus bioavailability due to intrinsic factors, such as age [33], race [34], sex [35], and/or genetic variations in cytochrome P450 3A and P-glycoprotein expression [36–38].

To our knowledge, the ELIT study is the first large, prospective, multicenter trial to investigate the impact of switching from IR-TAC or PR-TAC to LCPT on tremor in kidney transplant patients. The non-interventional design of the study is a strength, as the results reflect outcomes in standard clinical practice and therefore are generalizable to other clinical sites in France. However, the study does have a few limitations. Firstly, due to the observational nature of the study and the associated less stringent inclusion criteria, the study population was heterogeneous (e.g., the reasons for switching to LCPT and the TETRAS score at baseline were not set as inclusion criteria) and missing data may have limited the internal consistency of the results. Secondly, 65% of the population of the study was receiving corticosteroids as well as tacrolimus, which could have influenced tremor. Another limitation is that a subjective tremor assessment scale (TETRAS) was used rather than a more objective tremor assessment (such as accelerometers). However, in the study of patients in real-life conditions, using devices such as accelerometers is not practical, whereas TETRAS scores have been validated for use in this setting. The absence of a control group means that caution is required in the interpretation of the effect of LCPT treatment on tremor and health-related QoL. Furthermore, care is needed in the interpretation of the C_0_/D ratio improvement and its potential link with clinical outcomes. Therefore, the study results need to be confirmed in a randomized, controlled, international trial.

In conclusion, the results of the ELIT study suggest that LCPT could be beneficial to renal transplant patients. We observed an improvement in tacrolimus-induced tremor, as assessed with the TETRAS scale. Treatment with LCPT was also associated with a reduction in the daily dose of tacrolimus, while allowing a therapeutic trough blood concentration to be maintained. There was a trend towards improvement in other neurological symptoms, as well as significant improvements in patient health-related QoL. Further exploration of the pathophysiology of CNI-related toxicities and robust clinical investigations to fully discern the improved tolerability with LCPT are warranted.

## Data Availability

The raw data supporting the conclusion of this article will be made available by the authors, without undue reservation.

## APPENDIX 1

### The ELIT study investigators

Gabriel Choukroun (CHU Amiens Picardie-Site Sud, Amiens, France); Coralie Poulain, Maité Jaureguy, Hakim Mazouz (CHU Amiens Salouël, Amiens, France); Agnes Duveau, Julien Demiselle, Maud Cousin, Anne Sophie Garnier, Virginie Besson, Jean Francois Augusto, Jean-Francois Subra (CHU Angers, Angers, France); Isabelle Etienne, Niels Bruckmann, Audrey Dumont, Charlotte Laurent, Dominique Bertrand (CHU Rouen Hôpital de Bois Guillaume, Bois-Guillaume, France); Karine Moreau, Lionel Couzi, Benjamin Taton, Delphine Morel, Pierre-Gilles Merville (CHU Bordeaux Pellegrin, Bordeaux, France); Yannick Le Meur (CH Brest La Cavale Blanche, Brest, France); Nicolas Bouvier (CHU Caen, Caen, France); Anne-Elisabeth Heng (CHU Clermont Ferrand-G.Montpied, Clermont-Ferrand, France); Philippe Grimbert, Vincent Audard, Camille Petit Hoang, Djillali Sahali, Thomas Stehle, Khalil El Karoui, Marie Matignon, Philippe Remy (AP-HP Hôpital Henri Mondor, Creteil, France); Paolo Malvezzi, Lionel Rostaing, Benedicte Janbon (CHU Grenoble Alpes- Grenoble, France); Antoine Durrbach, Anne Grunenwald, Edouard Lefevre, Severine Beaudreuil (AP-HP Hôpital Le Kremlin Bicetre, Le Kremlin Bicetre, France); Jean Philippe Rerolle (Hôpital Dupuytren, Limoges, France); Valerie Moal, Raj Purgus, Tristan Legris (AP-HM Hôpital La Conception, Marseille, France); Valérie Garrigue, Georges Mourad, Vincent Pernin (CHRU Montpellier Hôpital Lapeyronie, Montpellier, France); Jacques Dantal, Diego Cantarovich, Magali Giral, Claire Garandeau (CHU Nantes-Hotel Dieu, Nantes, France); Laetitia Albano, Thierry Wine, Elisabeth Cassuto Viguier, Ahmed Jeribi (Chu Nice Hôpital Pasteur, Nice, France); Benoit Barrou, Maud Cazenave (AP-HP Hôpital Pitie Salpetriere, Paris, France); Christine Randoux, Quentin Rimbourg, Guillaume Hanouna, Latifa Azeroual, Caroline Du Halgouet, Prisca Mutinelli, François Vrtovsnik (AP-HP Hôpital Bichat, Paris, France); Alexandre Hertig, Eric Rondeau, Laurent Mesnard, Arezki Adem, Yosu Luque, Xiao Li Xu (Hôpital Tenon AP-HP, Paris, France); Dany Anglicheau, Olivier Aubert, Christophe Legendre, Claire Tinel, Rebecca Sberro Soussan, Franck Martinez, Julien Zuber, Pierre Tremolieres, Lynda Berehri, Anne Scemla, Lucile Amrouche, Alexandre Loupy, Gauthier Flahaut, Celine Estournet (AP-HP Hôpital Necker, Paris, France); Radia Choukria Allal (CH Rene Dubos, Pontoise, France); Leonard Golbin, Cecile Vigneau (CHU Rennes Pontchaillou, Rennes, France); Christophe Mariat, Ingrid Masson, Miriana Dinic, Nicolas Maillard, Christian Broyet, Guillaume Claisse, Eric Alamartine, Catherine Sauron, Hesham Mohey, Damien Thibaudin (CHU Hôpital Saint-Etienne, Saint-Priest-En-Jarez, France); Bruno Moulin, Noelle Cognard, Jerome Olagne, Laura Braun-Parvez, Francoise Heibel, Gabriela Gautier, Peggy Perrin, Sophie Caillard Ohlmann (Nouvel Hôpital Civil- Hôpital Universitaire de Strasbourg, Strasbourg, France); Nassim Kamar, Olivier Cointault, Laure Esposito, Arnaud del Bello, Anne-Laure Hebral (CHU Toulouse—Hôpital Rangueil, Toulouse, France); Matthias Buchler, Helene Longuet (CHRU Tours—Hôpital Bretonneau, Tours, France).
